# Association of knowledge, preventive counseling and personal health behaviors on physical activity and consumption of fruits or vegetables in community health workers

**DOI:** 10.1186/s12889-015-1643-3

**Published:** 2015-04-09

**Authors:** Alex A Florindo, Ross C Brownson, Gregore I Mielke, Grace AO Gomes, Diana C Parra, Fernando V Siqueira, Felipe Lobelo, Eduardo J Simoes, Luiz R Ramos, Mário M Bracco, Pedro C Hallal

**Affiliations:** School of Arts, Sciences and Humanities, University of São Paulo, Rua Arlindo Bettio, 1.000, CEP: 03828-000 São Paulo, SP Brazil; Prevention Research Center in St. Louis, Brown School, Division of Public Health Sciences and Alvin J. Siteman Cancer Center - Washington University in St. Louis, Missouri, USA; Postgraduate Program in Epidemiology, Federal University of Pelotas, Pelotas, RS Brazil; Department of Gerontology, Federal University of São Carlos, São Carlos, SP Brazil; Program in Physical Therapy Surgery (Prevention and Control), School of Medicine, Washington University in St. Louis, St. Louis, MO USA; School of Physical Education, Federal University of Pelotas, Pelotas, RS Brazil; Hubert Department of Global Health, Emory University – Rollins School of Public Health, Atlanta, USA; Department of Health Management and Informatics, School of Medicine, University of Missouri, Missouri, USA; Department of Preventive Medicine, Federal University of Sao Paulo, São Paulo, SP Brazil; Hospital Israelita Albert Einstein; Hospital Municipal Dr. Moysés Deutsch - M’Boi Mirim, São Paulo, SP Brazil

**Keywords:** Physical activity, Consumption of fruits and vegetables, Community health workers, Primary health care settings, Knowledge, Preventive counseling, Personal behavior

## Abstract

**Background:**

There is evidence that if a health professional is active and has a healthy diet, he/she is more likely to advise patients about the benefits of physical activity and healthy eating The aims of this study were to: (1) describe the personal physical activity, consumption of fruits and vegetables behaviors and nutritional status of community health workers; (2) evaluate the association between knowledge, delivery of preventive counseling and personal behaviors among community health workers.

**Methods:**

This was a cross-sectional study conducted in a nationally sample of health professionals working in primary health care settings in Brazil in 2011. This survey was part of the second phase of the Guide for Useful Interventions for Activity in Brazil and Latin America project, and data were collected through telephone interviews of 269 community health workers from the Unified Health Care system of Brazil. We applied questionnaires about personal reported behaviors, knowledge and preventive counseling in physical activity and consumption of fruits and vegetables. We calculated the prevalence and associations between the variables with logistic regression.

**Results:**

The proportion of community health workers that practiced 150 minutes per week of physical activity in leisure time or transportation was high (64.9%). Half of community health workers were overweight and only 26.2% reported consuming five portions/day of fruits or vegetables. Most community health workers reported counseling about physical activity for more than six months (59.7%), and most were not knowledgeable of the fruits and vegetables and physical activity recommendations. Meeting the fruits and vegetables recommendations was associated with correct knowledge (OR = 4.5; CI95% 1.03;19.7), with reporting 150 minutes or more of physical activity per week (OR = 2.0; CI95% 1.03;3.7) and with reporting physical activity in leisure time (OR = 2.0; CI95% 1.05;3.6). Regular physical activity counseling was associated with reporting 10–149 minutes per week (OR = 3.8; CI95% 1.1;13.3) and with more than 150 minutes of physical activity per week (OR = 4.9; CI95% 1.5;16.5).

**Conclusion:**

Actions to promote physical activity and healthy eating and to improve knowledge among community health workers within the health care system of Brazil could have a potential positive influence on delivery of preventive counseling to patients on this topic.

## Background

Physical inactivity, unhealthy diets, and obesity are among the top ten risk factors for morbidity and mortality worldwide [1]. This situation is particularly concerning in low and middle-income countries. In Brazil, for example, more than half of adult population is overweight, and 60% do not practice any kind of physical activity in leisure time, nor they do report regular consumption of fruits and vegetables [2,3]. A wide range of research and interventions have been implemented aimed at changing this scenario [4,5], but low and middle-income countries still have few policies implemented for promoting physical activity and fruits and vegetables intake [6]. In a recent study published by our research group, it was found that 39.8% and 72% of the primary care units in Brazil, develop any physical activity or healthy eating program, respectively [7].

The implementation of interventions in primary health care settings is a possible strategy for addressing the burden of obesity-related diseases [8]. Many of these interventions have focused on the training of health professionals, mainly physicians and nurses, to provide counseling for physical activity and healthy eating. Such interventions have shown positive results at improving health behaviors of patients, [9,10] and are facilitated by improving knowledge about the benefits of health promotion and improving counseling skills among health professionals in the primary care setting [10]. In addition, studies have shown that if the health professional is active and has a healthy diet, he/she is more likely to advise patients about the benefits of physical activity and healthy eating [11-15].

Most studies on this topic have been conducted with physicians and nurses [9-13]. In the last 10 years, community health workers have been shown to also play a key role in health promotion and disease prevention [16-25]. In the Brazilian context, community health workers living and working in the same neighborhood are typically responsible for visiting families in their homes in order to provide more personal guidance and counseling for disease prevention and health promotion, orientation about health services, and strengthen the linkage between health professionals teams and families. The health family team includes a physician, a nurse, auxiliary-nurses and a community health worker [26]. The Brazilian Ministry of Health has a guide that includes proposals of actions for the community health workers in Brazilian municipalities. This document contains orientations of counseling in healthy food, physical activity and weight control for health [27]. However, there is a lack of information about counseling and behaviors in physical activity, healthy food and obesity/weight control among community health workers, as well as associations between these factors. Therefore, the aims of this study were to: (1) describe the behaviors of physical activity, consumption of fruits and vegetables, and nutritional status of community health workers; (2) evaluate the associations between recommendations and knowledge of healthy behaviors (physical activity, fruits and vegetables consumption and body mass index) with personal healthy behaviors (fruits and vegetables consumption, body mass index and physical activity) among community health workers.

## Methods

We conducted a cross-sectional study with a nationally-representative sample of primary health care units in Brazil in 2011. This survey was part of second phase of the GUIA project in Brazil (Guide for Useful Interventions for Activity in Brazil and Latin America). We drew a random sample in three stages: (1) 1,600 primary health care units were selected randomly of a total 42,486 units in Brazil; (2) within the 1,600 sampled primary health care units, we listed three types of professionals: physicians, nurses and community health workers; (3) in 1/3 of the primary health care units we tried to interview the physicians, in 1/3 the nurses and in 1/3 the community health workers. A detail description of the sampling strategy of primary health care units and health professionals is available elsewhere [28]. We used random digit dialing survey to interview community health workers between January and July of 2011. We were able to interview one person in 1,279 primary health care units (79.9% of the approached ones). We interviewed 269 community health workers since only in one third of the units, the community health worker was the professional chosen. In addition, several of the units did not have a community health worker, thus the response rate of 50.5% should be interpreted with caution. The questionnaire was applied by telephone and included sociodemographic variables (age, gender, education, skin color, marital status), smoking, self-rated health, self-reported behaviors such as physical activity practice and consumption of fruits and vegetables, nutritional status, knowledge and professional conduct in physical activity, knowledge in consumption of fruits and vegetables and body mass index for classification of nutritional status.

### Variable description

#### Physical activity

We assessed the domains of leisure and transportation physical activity through self-report using the long version of the International Physical Activity Questionnaire (IPAQ). A validation study of this version of IPAQ administered by both face-to-face and telephone interviews of adults in Brazil is published [29]. We classified self-reported physical activity based on the recommendation of the World Health Organization [30]: a cutoff of 150 minutes per week of moderate-to-vigorous physical activity was applied, summing time spent active in the leisure time and transportation domains: 1) Physically active: practice of 150 minutes or more per week; 2) Insufficiently active: practice of 10 to 149 minutes per week; 3) Inactive: < 10 minutes per week. We also categorized practice of 150 minutes or more per week (yes or no); practice of leisure time physical activity (yes or no) and practice of transportation physical activity (yes or no).

### Consumption of fruits and vegetables

We used two questions to classify self-reported consumption of fruits and vegetables: (1) How many portions of fruits the community health workers consumed per day on average; (2) How many portions of vegetables the community health workers consumed per day on average. If the interviewee had doubts about the meaning of one portion, the interviewer used examples (speaking of an apple or banana for example). Natural juices and vegetable soups were also included. We classified community health workers consuming at least five portions per day of fruits or vegetables as having a healthy diet according to the World Health Organization recommendation or otherwise [31].

### Body mass index

We calculated body mass index (BMI) based on self-reported weight and height. We categorized BMI using the World Health Organization standards [31]: (1) normal weight: BMI ≤ 24.9 kg/m^2^; (2) overweight: BMI ≥ 25.0 kg/m^2^; (3) obesity: BMI ≥ 30.0 kg/m^2^.

### Knowledge about physical activity, consumption of fruits and vegetables and nutritional status

We assessed knowledge about physical activity, consumption of fruits and vegetables, and nutritional status based on the recommendations from the World Health Organization [31], according to the following criteria:

1. Physical activity: We used two open questions, community health workers could select the answer that they considered to be correct: (1) Days per week of practice of moderate-intensity physical activity needed to achieve health benefits. The answer considered as adequate was a minimum of five days; (2) Time per day of practice of moderate-intensity physical activity needed to achieve health benefits. The answer considered as adequate was a minimum of 30 minutes per day. We classified knowledge of physical activity as a practice of at least 30 minutes per day of moderate activities in at least five times per week. This questionnaire has been used in one intervention study with community health workers in Brazil [32].
2. Consumption of fruits and vegetables: We used one closed question of multiple choice about the minimum of portions of fruits and vegetables that an adult should consume per day with. The question had seven options. The correct choice was a minimum of five portions per day of fruits or vegetables.
3. Weight status: We applied one multiple choice closed question regarding cutoff points for overweight and obesity. The question had five options. The correct choice was BMI ≥ 25.0 kg/m^2^ for overweight and BMI ≥ 30.0 kg/m^2^ for obesity.

### Professional advice/counseling for promoting physical activity

We used one question about community health workers’ habit to give advice about physical activity to patients, with six possible answers: (1) Does not advise and does not plan to start; (2) Does not advise, but does intend to start; (3) Does advise, but not regularly; (4) Does advise, but started recently; (5) Advises regularly, for more than six months; (6) Used to advise, but stopped doing so. We categorized professional practice for community health workers that positive response to option 5 (advises regularly, for more than six months). This questionnaire was already used in an intervention study among community health workers in Brazil [32].

### Data analysis

We measured the prevalence of community health workers’ self-reported physical activity, consumption of fruits and vegetables and nutritional status; their preventive counseling for physical activity; and knowledge about recommendation for physical activity, knowledge about recommendation for consumption of fruits and vegetables and the knowledge about cutoff for classification of overweight and obesity. Initially we calculated the bivariate association between all variables using chi-square test: 1) leisure time physical activity (yes or no); 2) transportation physical activity (yes or no); 3) practice of at least 150 minutes of physical activity per week (yes or no); 4) practice of physical activity in three levels (practice of 150 minutes or more per week; practice of 10 to 149 minutes per week; inactive or < 10 minutes per week); 5) consumption of at least five portions per day of fruits or vegetables (yes or no); 6) BMI (≥25.0 kg/m^2^ or ≤ 24.9 kg/m^2^); 7) physical activity counseling for more than six months (yes or no); 8) knowledge about physical activity recommendations (know or not know); 9) knowledge about the recommendations of fruits and vegetables consumption (know or not know); 10) knowledge about cutoff of BMI for classification overweight and obesity (know or not know). All variables that had significant association for p < 0.05 were calculated logistic regression models and odds ratio, unadjusted and adjusted by gender, age and education. The selection of dependent variables for regression models was done based in two criteria: 1) variables that were more associated between all of them; 2) variables that were dichotomized. All analysis were performed using SPSS software, version 15.0.

### Ethical issues

The study was approved by the Ethics Committee of the Federal University of Pelotas (process number 16154).

## Results

**Figure 1:**
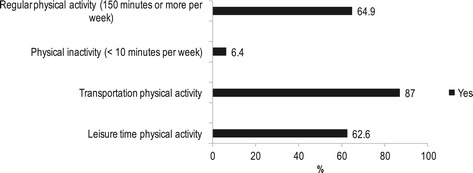
**Prevalence of reported practice of physical activity in leisure time (yes or no, n = 265), transportation (yes or no, n = 254), 150 minutes per week of leisure time or transportation (yes or no, n = 251) and physical inactivity (yes or no, n = 251) in community health workers, Brazil, 2011.**

**Figure 2:**
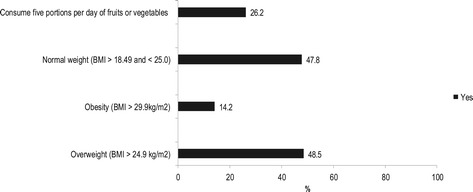
**Nutritional status based body mass index (BMI, n = 268) and consumption of fruits or vegetables (five portions per day, yes or no, n = 267) in community health workers, Brazil, 2011.**

The respondents in our sample of community health workers were mainly women, ages 49 years and older, had less than 11 years of education, had non-white skin color, were married or living with a partner, had good or very good health, and were not smokers (Table 1). Most community health workers practiced leisure time or transportation physical activity and achieved physical activity recommendations for health (Figure 1), but did not consume five portions per day of fruits or vegetables. Half of them were overweight (Figure 2).

**Table 1:** **Sociodemografic and general health characteristics of community health workers, Brazil, 2011**

**Variables**	**Community health workers %**
***Gender***	
Men	10.8
Women	89.2
***Education (years of study)***	
Up to 8 years	7.8
9 to 11 years	79.6
12 years or more	12.6
***Age***	
18 to 29 years old	28.3
30 to 49 years old	61.3
50 years or more	10.4
Skin Color*	
White	39.0
Non-white	61.0
***Marital status***	
Single	24.2
Married/Living with partners	66.9
Widowed/Divorced/Separated	8.9
***Self-report of health***	
Bad or very bad	20.1
Good or very good	79.9
***Smoking***	
Yes	7.4
No	92.6

*2 missing.

**Figure 3:**
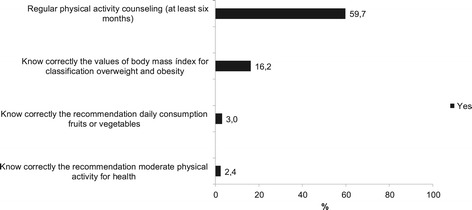
**Professionals conduct about regular physical activity counseling (yes or no, n = 268), knowledge about cutoff of BMI for classification overweight and obesity (know or not know, n = 268), knowledge about the recommendations of fruits and vegetables consumption (know or not know, n = 268), and knowledge about physical activity recomendations (know or not know, n = 247) in community health workers, Brazil, 2011.**

Most community health workers were not aware of the recommendations of physical activity and consumption of fruits and vegetables for health as well as BMI cut off values for the classification of overweight and obesity. Over half of community health workers reported advising physical activity to people of their covered area (Figure 3).

Five significant associations with p < 0.05 were identified from bivariate analysis: (1) consumption of at least five portions per day of fruits or vegetables (yes or no) was associated with knowledge about the recommendations of fruits and vegetables consumption (know or not know) (p = 0.018); (2) leisure time physical activity (yes or no) was associated with consumption of at least five portions per day of fruits or vegetables (yes or no) (p = 0.023); (3) practice of at least 150 minutes of physical activity per week (yes or no) was associated with consumption of at least five portions per day of fruits or vegetables (yes or no) (p = 0.038); (4) practice of physical activity in three levels (practice of 150 minutes or more per week; practice of 10 to 149 minutes per week; inactive or < 10 minutes per week) was associated with consumption of at least five portions per day of fruits or vegetables (yes or no) (p = 0.018); (5) practice of physical activity in three levels (practice of 150 minutes or more per week; practice of 10 to 149 minutes per week; inactive or < 10 minutes per week) was associated with physical activity counseling for more than six months (yes or no) (p = 0.011).

We opted to put the consumption of at least five portions per day of fruits or vegetables (yes or no) and physical activity counseling for more than six months (yes or no) as dependent variables in logistic regression models, producing unadjusted and adjusted odds ratios (Table 2). Knowledge about recommendations for consumption of fruits and vegetables, just as practice of leisure time physical activity and the practice of regular physical activity for health were associated with higher odds ratios for consumption of five portions per day of fruits or vegetables. The practice of some physical activity or according to recommendations for health was associated with higher odds ratio for physical activity counseling. While three-level physical activity was associated with consumption of five portions of fruits or vegetables in bivariate analysis (p-trend), this association disappeared after adjustment in logistic models.

**Table 2:** **Relationships of knowledge about healthy behaviors and preventive counseling for physical activity self-reported healthy behaviors among community health workers, Brazil, 2011**

	**Reported consuming five portions per day of fruits or vegetables**	
	**OR (CI95%)** ^**1**^	**OR (CI95%)** ^**2**^
*Correct knowledge of daily fruits and vegetables to consumption recommendation (five portions per day)*		
No	1	1
Yes	5.0 (1.2 – 21.4)	4.5 (1.03 – 19.7)
*Reported physical activity (minutes per week)*		
<150 minutes	1	1
150 minutes or more	2.0 (1.03 – 3.7)	2.0 (1.03 – 3.7)
*Leisure time physical activity reported*		
No	1	1
Yes	2.0 (1.1 – 3.7)	2.0 (1.05 – 3.6)
*Reported physical activity (minutes per week)*		
Physical inactivity (<10 minutes)	1	1
Practice of 10 to 149 minutes	4.0 (0.5 – 32.3)	4.0 (0.5 – 33.0)
Practice of 150 minutes or more	6.5 (0.8 – 50.6)	6.5 (0.83 – 51.2)
	**Counseling on regular physical activity (at least six months) (yes)**	
	**OR (CI95%)** ^**1**^	**OR (CI95%)** ^**2**^
*Reported physical activity (minutes per week)*		
Physical inactivity (<10 minutes)	1	1
Practice of 10 to 149 minutes	3.4 (1.0 – 11.8)	3.8 (1.1 – 13.3)
Practice of 150 minutes or more	4.7 (1.4 – 15.5)	4.9 (1.5 – 16.5)

OR (odds ratio); CI (confidence interval) ^1^Unadjusted; ^2^Adjusted for gender, age and education.

## Discussion

This study showed that healthy behaviors reported, such as physical activity and consumption of fruits and vegetables, were associated with knowledge and preventive counselling among community health workers in Brazil. To our knowledge, this is the first study that showed that physical activity practice was associated with physical activity counseling among community health workers.

Health promotion interventions that include community health workers are important because these professionals have the potential to influence the behavior of patients from having a closer contact with them within the primary health care system [16-24]. Although most community health workers practice physical activity at recommended levels, most do not consume fruits and vegetables in accordance with recommendations, and half were overweight. In comparison with the Brazilian adult population, community health workers practice more physical activity, but the consumption of fruits and vegetables as well as overweight and obesity prevalence were similar [2,3]. Interventions to improve health promotion of the community health workers can result in more effective professional actions for patients and the whole population who are covered by them. A controlled study conducted with health professionals in Israel showed that an intervention for physicians and dietitians resulted in significant reduction of waist circumference and improved knowledge for counseling about weight loss (physicians) and healthy lifestyle (dietitians) [33]. In the same study, nurses and health promotion specialists did not change physical activity levels or healthy eating behaviors or showed reduction in waist circumference, which shows the complexity of changing behaviors [33]. A systematic review conducted with physicians and nurses showed that the association between weight status and management practices for weight control in patients is weak [34]. In our study with community health workers, we also found no association between knowledge about the correct classification of overweight or obesity and BMI. Four years of intervention conducted by Frank et al. to increase physical activity, consumption of fruits and vegetables and cessation of smoking and alcohol consumption among medical students, resulted in better preparation for counseling for physical activity and healthy eating among these students [15].

This study showed a high prevalence of community health workers who did not know the recommendations of physical activity and consumption of fruits and vegetables for health. Half these community health workers were overweight and most of them do not consumed five portions of fruits and vegetables per day. Therefore, interventions to improve knowledge and health behaviors related to physical activity and healthy eating are needed among community health workers.

Other studies have shown that physicians and nurses of primary health care settings in Brazil [28] as well as general practitioners, practice nurses and health visitors in Scotland did not know the recommendations of physical activity for health [35]. We have evidence that interventions can improve knowledge and professional conduct among community health workers [32,36] and they are similar among other health professionals [15,37]. Community health workers are more usually women of lower socioeconomic status. These professionals and others who are in stressful jobs (prone to burnout and poorer health) are sometimes overlooked in interventions to promote health [38,39]. Therefore, interventions to improve lifestyle of community health workers are needed.

The results of this study showed that although the prevalence of physical activity counseling was high, community health workers had little knowledge about a recommendation or global strategy for physical activity promotion and healthy diet of the World Health Organization. The high prevalence of physical activity counseling is possibly influenced by physical activity promotion policies implemented by the Brazilian Ministry of Health during the last years and encouraged in cities and health care services for health promotion [40,41]. But the little knowledge about physical activity indicates that community health workers need a more organized and comprehensive training. We believe that there is not a standard for community health workers to advise in physical activity in primary health care system in Brazil. For example, the practice guide of community health workers [27] needs to be improved with better information about physical activity promotion and the Brazilian Ministry of Health could encourage the municipalities in training these professionals. Some models of training for physical activity promotion by community health workers has been studied by researchers in Brazil and could be applied [32,42]. This could contribute in actions to fight chronic diseases for reduce physical inactivity [43].

The study has some limitations. The cross-sectional data does not allow us to determine temporality and thus move toward establishing. The methods for evaluation of knowledge were not validated against “gold standards” (e.g., for preventive counseling). A study published recently among community health workers used a similar questionnaire to evaluate the knowledge about physical activity showing that this instrument was adequate for evaluating changes in physical activity promotion [32]. Another limitation is the high non-response rate of 50.5% and the missing information about community health workers in primary health care units. Not all primary health care units had community health workers, because professionals in some units use a different model (the health family model). This fact may have introduced bias toward a non–representative sample. In addition, these professionals may have had time constraints for response and lack of telephone lines in some primary health care units. However, the sample of this study is very similar to other samples of community health workers in Brazil in relation to age, education and gender [44-46], minimizing the possibility of selection bias.

## Conclusions

This study showed that most community health workers in Brazil practice physical activity and advise their patients to physical activity, but do not know the recommended amount of physical activity needed to obtain health benefits. Most community health workers did not know recommendations of the consumption of fruits and vegetables, did not consume at least five portions per day of fruits and vegetables, and half are overweight. We found an association between knowledge, preventive counselling and healthy behaviors reported of physical activity and consumption of fruits and vegetables. Therefore, actions to health promotion are needed for community health workers. On the other hand, it seems promising that community health workers can disseminate physical activity and healthy eating counseling into the communities where they serve, with appropriate training.
